# Extramammary Paget’s Disease: Diagnosis, Pathogenesis, and Treatment with Focus on Recent Developments

**DOI:** 10.3390/curroncol28040260

**Published:** 2021-08-05

**Authors:** Shoichiro Ishizuki, Yoshiyuki Nakamura

**Affiliations:** Department of Dermatology, Faculty of Medicine, University of Tsukuba, Tsukuba 305-8575, Japan; syoichiro200711668@gmail.com

**Keywords:** Extramammary Paget’s disease, diagnosis, treatment, recent developments

## Abstract

Extramammary Paget’s disease (EMPD) is a rare neoplasm that usually develops in apocrine gland-bearing areas, such as the vulva, scrotum, and penis. EMPD may present with a focal, multifocal, or an ectopic lesion. Clinically, EMPD lesions often exhibit infiltrative erythema, which is sometimes similar to other skin disorders such as eczema. While primary EMPD arises as intraepithelial neoplasm of the epidermis, EMPD-like lesions may occur from epidermotropic spread of malignant cells or direct extension from an underlying internal neoplasm, known as secondary EMPD. Because treatment strategies differ for primary EMPD and secondary EMPD, accurate diagnosis based on detailed histopathological evaluation is required. In the early stages, EMPD usually shows indolent growth, and most cases are diagnosed as carcinoma in situ. However, invasive lesions may result in metastases, and deep invasion is associated with high incidence of metastases. Conventional chemotherapies have been used for EMPD treatment in patients with distant metastases, but the efficacy is not satisfactory, and the prognosis for such patients remains poor. Recent studies have provided various insights into the molecular pathogenesis of the development and advancement of EMPD, which may lead to novel treatment approaches for metastatic EMPD. This review addresses the diagnosis, pathogenesis, and treatment of EMPD with focus on recent progress in understanding this disease.

## 1. Introduction

Extramammary Paget’s disease (EMPD) is a rare neoplasm that usually develops in the apocrine gland-bearing areas of older adults [1,2,3,4]. Clinically, EMPD lesions often exhibit infiltrative erythema with crust and scale, sometimes resembling other skin disorders such as eczema. EMPD can be classified into primary and secondary EMPD manifestations. While primary EMPD arises as intraepithelial neoplasm of the epidermis, secondary EMPD develops from epidermotropic spread of malignant cells or direct extension from an underlying internal neoplasm [5,6]. However, it is sometimes difficult to distinguish primary EMPD and secondary EMPD both clinically and histologically. As the treatment strategy and prognosis for primary and secondary EMPD differ, accurate diagnosis based on detailed histopathological evaluation of multiple immunohistochemical markers is required. Most EMPD cases are diagnosed as carcinoma in situ, which usually shows indolent disease progression. However, once Paget cells invade deeply into the dermis, regional lymph node (LN) metastases and distant metastases frequently develop [7]. Cases with distant metastases have a poor prognosis because conventional chemotherapies, traditionally used for EMPD treatment, show limited efficacy. Recent studies have provided various insights into the mechanism and associated molecules of EMPD development and advancement, which may lead to novel treatment approaches for metastatic EMPD. Here, focusing on recent developments, we review EMPD, including its diagnosis, pathogenesis, and treatment.

## 2. Review

### 2.1. Epidemiology

EMPD is rare, and a recent study showed that the crude prevalence of EMPD in mainland China was 0.4 per 1,000,000 people in 2016, which is a similar ratio to the age-adjusted prevalence [8]. In 2012, van der Zwan et al. reported that the crude incidence rate and European age standardized incidence rate of invasive EMPD within Europe was 0.7 and 0.6 per 1,000,000 person-years, respectively [9]. The prevalence of EMPD regarding gender differs between Western and Asian populations. In studies of Caucasian populations, EMPD has a female predominance, with male-to-female ratios ranging from 1:2 to 1:7 [1,9,10,11,12]. In contrast, male predominance has been reported in Asian studies [3,13]. A multicenter institution study conducted in Japan revealed that 327 (60.1%) of 544 patients were male [3], and a nationwide population-based study in Taiwan revealed that the male-to-female ratio among EMPD patients was 3.5:1 [13]. EMPD usually develops in older adults, with the mean age at diagnosis being 60–70 years [3,4].

### 2.2. Clinical Presentation

According to Ghazawi et al., the predominant sites of EMPD development are the scrotum and penis in males and the vulva in females [3]; however, perianal, axillar, or umbilicus regions are also sometimes involved. EPMD can be multifocal, and cases of EMPD developed in more than 2 apocrine gland-bearing areas, known as double, triple, or synchronous EMPD, have also been reported [14,15]. Therefore, a thorough physical examination of these regions is important when EMPD is encountered. EMPD skin lesions typically present as a well-circumscribed erythema or erythematous plaques with occasional hyperpigmentation or hypopigmentation. Cases in which the main clinical presentation was hypopigmentation have also been reported, and such cases are difficult to clinically diagnose as EMPD [16]. Crust, scale, or erosion on the erythematous lesions may also be seen, and such lesions may mimic various other skin disorders, such as eczema, psoriasis, and fungal infection. In the later stages, nodules or deep ulcers may occur. Although associated symptoms, such as pruritus and tenderness, may develop, around 10% of EMPD patients are asymptomatic [17,18]. EMPD lesions sometimes show subclinical extension, making it difficult for clinicians to determine the clinical borders between EMPD lesions and the surrounding normal skin [19].

Dermoscopy is a non-invasive tool for diagnosis of skin lesions and has been shown to improve clinical diagnosis of melanocytic lesions [20,21]. Recently, dermoscopic features of EMPD lesions have also been investigated [22,23]. Mun et al. compared dermoscopic features of EMPD lesions and other skin lesions, the gross findings of which may be similar to EMPD, including eczema, fungal infection, and Bowen disease [22]. They found that, among the dermoscopic findings common to other skin lesions, such as milky-red areas, dotted vessels, glomerular vessels, polymorphous vessels, surface scales, and linear irregular vessels, milky red areas were significantly more frequent in EMPD than in eczema, fungal infection, and Bowen disease [22]. In addition, vascular structures were also more common in EMPD than in eczema and fungal infection [22]. In contrast, Payapvipapong et al. suggested that the lava lake structure, defined as a combination of white, branching reticular lines and intermingling white clods resembling a lava lake inside a live volcanic crater, and the cloud-like structureless area, defined as a combination of a white structureless area resembling diffuse layering of stratus clouds and small, round, white clods resembling fluffy cumulus clouds, may be specific dermoscopic findings of EMPD [23].

### 2.3. Classification

Primary EMPD is defined by lesions that initially develop as intraepithelial neoplasm of the epidermis. Although primary EMPD typically develops in apocrine gland-bearing areas, it rarely develops in non-apocrine gland-bearing areas such as the face, back, arms and legs [5,6]. Cases of multiple ectopic EMPD have also been reported [24]. Ectopic EMPD appears to be more common among Asian patients [6]. There is no clinical or histological difference between ectopic EMPD and classic (non-ectopic) primary EMPD except for the location [5,6]. Secondary EMPD is defined by primary EMPD-like lesions that develop from the epidermotropic spread of malignant cells or direct extension from an underlying internal neoplasm, such as colorectal carcinoma and urothelial carcinoma [6,25]. Because the treatment strategy and prognosis for primary secondary EMPD differ, accurate diagnosis based on detailed histopathological evaluation is required. Previously, Wilkinson et al. proposed vulvar EMPD classification as either primary or secondary EMPD, with three subtypes for each classification [26]. Primary EMPD is subdivided into intraepithelial cutaneous Paget disease in situ as the usual type, intraepithelial cutaneous Paget disease with invasion, and intraepithelial cutaneous Paget disease as a manifestation of underlying skin-appendage adenocarcinoma [26]. Secondary EMPD is subdivided into Paget disease of anorectal origin, Paget disease of urothelial origin, and Paget disease of other origin [26]. This proposed classification is useful to organize the various types of EMPD and avoid potential confusion (Table 1). 

### 2.4. Histopathology

Histopathological examination of EMPD reveals the presence of Paget cells in the epidermis, which are characterized by atypical large cells with abundant, clear, and sometimes eosinophilic cytoplasm in hematoxylin and eosin staining [27]. The cells are present singly or form clusters. In some cases, distinction from Bowen’s disease or melanoma can be difficult without use of immunohistochemical studies [28]. Tumor cells may be pigmented, or there can be colonization of the involved epidermis by non-neoplastic dendritic melanocytes [29]. The epidermis of EMPD often shows acanthosis with hyperkeratosis, parakeratosis, or ulceration [30]

Paget cells, irrespective of whether primary or secondary EMPD, are usually positive for the diastase-periodic acid-Schiff (PAS) reaction, mucicarmine, and zirconyl hematoxylin, indicating the presence of neutral mucopolysaccharides. Immunohistochemical studies are essential for accurate diagnosis of EMPD, especially for differentiating primary EMPD from secondary EMPD. Secondary EMPD frequently develops from colorectal carcinoma and urothelial carcinoma [31]. Primary EMPD is positive for CEA, with a positive rate of 84.2–98.9% [32,33]. However, both colorectal carcinoma and urothelial carcinoma may also show positive staining for CEA [34,35]. The most useful marker for excluding the possibility for secondary EMPD arising from colorectal carcinoma and urothelial carcinoma is gross cystic-disease fluid protein-15 (GCDFP-15), which is usually negative for these lesions, whereas GCDFP-15 is sometimes positive for primary EMPD. However, the positive rate of GCDFP-15 in primary EMPD has been reported to be as little as 30.0–52.6%, and substantial cases of primary EMPD show negative staining of GCDFP-15 [32,36,37]. The combination of CK7 and CK20 staining is also crucial in distinguishing primary EMPD and secondary EMPD [31]. Whereas primary EMPD typically shows CK7^+^/CK20^-^, secondary EMPD from colorectal carcinoma typically shows CK7^-^/CK20^+^. However, although relatively rare, cases of primary EMPD with positive CK20 staining and cases of secondary EMPD developed from colorectal carcinoma with positive CK7 or negative CK20 staining have also been reported [38,39,40]. Meanwhile, secondary EMPD arising from urothelial carcinoma typically shows CK7+/CK20+ [31,41]. However, cases of secondary EMPD developed from urothelial carcinoma showing negative CK20 staining have also been reported [42], suggesting that the combination of CK7/CK20 staining is not an absolute method to discriminate primary from secondary EMPD. As for other markers, CDX-2, an intestinal cell marker, and uroplakin II and III, urothelial cell markers, could be also useful for the discrimination: both are usually negative for primary EMPD [40,43,44,45]. Collectively, comprehensive evaluation using multiple markers, including CK7/20, GCDFP-15, CDX-2, and uroplakin II and III, is required for accurate discrimination of primary EMPD and secondary EMPD. The microscopic morphology and distribution of tumor cells in melanoma and Bowen disease also sometimes resemble EMPD, and cases of Bowen disease resembling EMPD are known as Pagetoid Bowen disease [46,47]. Whereas melanoma cells are typically positive for S-100, HMB-45, and Melan-A, and atypical keratinocytes of Bowen disease are typically positive for p63, Paget cells in EMPD are usually negative for all of these molecules [47,48,49]. In addition, negative staining of PAS and CEA in melanoma and Bowen disease tumor cells also aid in distinguishing these diseases from EMPD. 

### 2.5. Pathogenesis

Some previous studies involving genomic analyses in EMPD lesions reported somatic mutations in various genes, including *TP53*, *ERBB*, *NRAS*, *BRAF*, *PIK3CA*, and *AKT1* genes [50,51]. Ishida et al. conducted genetic analyses of 87 EMPD lesions and their exome analysis identified *ERBB2*, *ERBB3*, *KMT2C*, *TP53*, *PIK3CA*, *NUP93*, *AFDN*, and *CUX1* as likely driver mutations [52]. Their copy-number alteration analysis showed regions spanning *CDKN2A* as recurrently deleted and *ERBB2* as recurrently amplified and that greater copy-number alteration load correlated with high frequency of recurrence [52]. Frequent gene alternations in *ERBB2*, *RAS*, *RAF*, *AKT1*, and *PIK3C* in EMPD lesions detected in previous studies suggested that HER2, which is encoded by *ERBB2*, and its downstream signaling, including RAS/RAF-MEK-ERK pathway and PI3K-AKT-mTOR pathway, may play important roles in the pathogenesis of EMPD in many cases. Takeichi et al. also analyzed genomic alteration in 48 patients with EMPD and identified *FOXA1* mutations, a *GAS6–FOXA1* fusion gene, and somatic hotspot mutations in the *FOXA1* promoter region in 11 of the 48 patients [53]. They also revealed that FOXA1 expression was strongly expressed in Paget cells in all of the EMPD samples and was associated with estrogen receptor (ER) expression [53]. Given that FOXA1 assists the transcriptional activity of the ER and may be involved cooperatively in the tumorigenesis of breast cancer, the FOXA1-ER axis may also play crucial roles in EMPD development and progression in some cases [54].

As for molecular expression in EMPD lesions, multiple studies have demonstrated HER2 overexpression in EMPD lesions, which was associated with amplified *ERBB2* [55,56]. These HER2-positive EMPD cases conferred a more aggressive biology [57]. In addition, Tanaka et al. demonstrated that around 90% of patients with EMPD showed no difference in HER2 overexpression and *ERBB2* gene amplification between primary lesions and lymph node (LN) metastasis, indicating that targeting therapies for HER2 may be effective for the treatment of both primary and metastatic lesions [58]. Lin et al. used microarray analysis and identified that expressions of *ERBB4*, *PRLR*, *TCF3*, *PIK3R3*, *SULT1A1*, and *TCF7L1* were significantly overexpressed in EMPD lesions [59]. Immunohistochemical studies also confirmed the overexpression of PRLR, a prolactin (PRL) transmembrane receptor, interacting with PRL to activate downstream signaling in breast cancer [59,60]. Therefore, targeting therapies for the PRL-PRLR axis might be an effective EMPD therapy. Although most EMPD cases exhibit low expression of estrogen receptor (ER), EPMD shows a high androgen receptor (AR)-positive rate at 54–90%, and AR expression was stronger in invasive EMPD than in situ EMPD [61,62,63], indicating that AR signaling may be involved in the EMPD progression and that blockade of AR signaling might be another effective therapy for EMPD. Chang et al. immunohistochemically evaluated expression of CXCR4 and CXCR7 in 92 EMPD lesions [64]. CXCR4 and CXCR7 share the same chemokine ligand, CXCL12, and have been reported to play important roles in tumor growth, angiogenesis, and metastases in various cancer, such as lung and breast cancer [64]. They demonstrated that high expression of CXCR4 and CXCR7 were both correlated with regional LN metastases and presence of lymphovascular invasion, and high expression of CXCR7 also correlated with the depth of invasion. Therefore, both CXCR4 and CXCR7 can be used as biomarkers for prediction of the aggressiveness of EMPD, and therapies targeting CXCR4 and CXCR7 may be helpful to prevent EMPD progression [64].

There have been multiple studies regarding the immune environment in EMPD. Regulatory T cells (Tregs), which show FoxP3 expression; suppress the activation of other immune cells, including effector T cells; and play a crucial role in immune evasion in various malignant tumors. A previous study demonstrated that the number of CD4^+^ cells and CD8^+^ cells as well as the CD8^+^/FoxP3^+^ ratio were significantly decreased in the epidermis of vulvar EMPD compared with healthy controls, whereas the stromal compartment was highly infiltrated by various immune cells, including T cells and myeloid cells, suggesting immunocompromised environment in the epidermis of EMPD lesions [65]. The authors speculate that the immune cells may be unable to penetrate the epidermis to clear Paget cells [65]. Press et al. analyzed FoxP3^+^ cells at the dermal–epidermal junction in vulvar EMPD and found that both nonrecurrent and recurrent cases with positive surgical margin had more FoxP3^+^ cells than those with negative surgical margin, suggesting that increased Tregs may be associated with more extensive EMPD cases, and these Tregs might promote survival and subclinical spread of Paget cells along the epidermis [66]. Macrophage are other immune cells that significantly regulate immune response to tumor. Fujimura et al. reported that large numbers of CD163^+^ M2 macrophages and metalloproteinase (MMP)-9^+^ cells were detected in invasive but not in situ EMPD lesions, suggesting that an increased number of MMP-9^+^ cells may be associated with CD163^+^ M2 macrophages and may be involved in the progression of invasive EMPD [67]. Given that aminobisphosphonate has been reported to decrease pro-MMP-9 and may abrogate the induction of CD163^+^ M2 macrophages in the tumor microenvironment, bisphosphonates might be effective for the prevention of not only bone metastasis but also disease progression in patients with invasive EMPD [68,69]. Receptor activator of nuclear factor κB ligand (RANKL) and its receptor RANK have multiple divergent effects in immunity; they not only activate T-cell priming but also promote Treg generation, which may induce tolerance against tumor antigens [70,71]. Kambayashi et al. demonstrated that expressions of RANKL and MMP-7, which cleaves RANKL to release a soluble form of RANKL (sRANKL), in EMPD lesions were higher than in non-lesional skin [72]. In contrast, RANK was mainly expressed by CD163^+^ Arg1^+^ CD206^+^ M2 macrophages, suggesting that the sRANKL released from Paget cells may stimulate these M2 macrophages via RANK [72]. It has also been reported that most of the CD163^+^ M2 macrophages expressed CCL17 in EMPD lesions [73]. CCL17 attracts CCR4-expressing cells, which are mainly T cells, and CCR4 is highly expressed in effector Tregs, characterized by high expression of FoxP3 [74]. Consistently, Foxp3^+^ cells have been shown to surround CD163^+^ M2 macrophages, indicating that M2 macrophages stimulated by RANKL/RANK signaling might recruit effector Tregs into the tumor microenvironment of EMPD [73]. Therefore, denosumab, which is an anti-RANKL antibody and is currently approved for use to treat bone metastases, could be a potential treatment for advanced EMPD lesions. 

Recent clinical trials have revealed that immune checkpoint inhibitors, including anti-PD-1/PD-L1 antibodies and anti-CTLA4 antibodies, improved survival of patients with various malignancies, such as melanoma and renal cell carcinoma [75,76,77]. PD-L1/PD-L2 expression is known to correlate with the anti-tumor response of anti-PD-1/PD-L1 antibodies [78,79,80]. However, Karpathiou et al. conducted immunohistochemical studies of 41 patients with EMPD and showed that PD-L1 was not expressed by any tumor cells or the associated lymphocytes, although dense T-cell infiltration was observed [81]. Pourmaleki et al. also demonstrated very focal expression of PD-L1 and no expression of PD-L2 in EMPD lesions [82]. They revealed high expression of B7 family members B7-H13 and B7-H14 in EMPD lesions, indicating that targeting therapies for these molecules might be effective for EMPD treatment [82]. In contrast, Mazuo et al. demonstrated that PD-L1 was expressed in tumor cells in 3 of 21 (14%) EMPD cases and in tumor-infiltrating immune cells in 15 of 21 (71%) EMPD cases [83]. They also showed that PD-1 was expressed in the tumor-infiltrating immune cells in all cases, although the density of PD-1 and PD-L1 in the tumor did not correlate with any clinical data, including overall survival (OS), disease-specific survival, and time to metastasis [83]. Recently, Kawaguchi et al. also revealed that PD-L1 and PL-L2 were expressed in tumor cells in 13 of 47 (27.7%) EMPD cases and 21 of 47 (44.7%) EMPD cases, respectively [84]. In this study, both PD-L1 and PD-L2 expression, as well as low CD8^+^ tumor-infiltrating T-cell (TIL) numbers, were associated with shorter postoperative recurrence-free survival, suggesting that the expression of PD-L1/PD-L2 in tumor cells may be a factor for worse prognosis [84]. The discrepancy of PD-L1 and PD-L2 expression between each study may be explained by the different cut-off values provided, differences in used antibody clones, and difference of the ratio of invasive cases [83,84]. It has been reported that the status of high microsatellite instability (MSI-H) is another predictor for better anti-tumor response from anti-PD-1 antibodies [85]. Although germline mutations in mismatch repair genes, such as MLH1 and MSH2, which may be associated with microsatellite instability, were found in some EMPD patients, previous studies have reported that MSI-H status was not observed in most EMPD cases [86,87]. A review for immune checkpoint inhibitors in vulvar neoplasms including EMPD was recently reported [88].

### 2.6. Patient Evaluation 

EMPD is associated with increased risk of other malignancies [3]. Ghazawi et al. demonstrated that 87 (16%) of 544 EMPD patients had at least one additional invasive malignancy, and more male patients with EMPD had association with additional malignancy [3]. Common sites of the associated malignancies were the colon, rectum, prostate, and bladder; therefore, consideration of screening for internal malignancies might be required when EMPD is diagnosed. However, a recent population study with age-matched control showed no increased risk of associated malignancies in noninvasive primary vulvar EMPD patients, indicating that risk of other malignancies have been overestimated in large elderly population of EMPD patients [89,90]. From this finding, the authors suggest that routine screening for other malignancies in patients diagnosed with primary noninvasive vulvar EMPD may not be needed.

As for the prognostic factors of EMPD, Ito et al. conducted a retrospective analysis of 35 EMPD patients and demonstrated that the presence of a nodule on the primary lesion, clinically palpable lymph nodes, the level of tumor invasion, and lymph node metastases were significant factors for the prognosis [91]. In contrast, Preti et al. analyzed 122 vulvar EMPD patients and reported that the cancer-specific survival at 120 months was 100% for intraepithelial and microinvasive (≤ 1 mm) vulvar EMPD but 31% for invasive (>1 mm) vulvar EMPD [92]. Similarly, van der Linden et al. analyzed 113 patients with vulvar EMPD and demonstrated that the 5-year disease-specific survival rate was greater than 98% in noninvasive and microinvasive EMPD but was only 50% and significantly worse in invasive EMPD. Therefore, microinvasion may not significantly affect the prognosis of EMPD patients [93]. 

Sentinel lymph node biopsy (SLNB) is a surgical procedure used to determine whether primary tumor has spread to regional LNs. The role of SLNB in EMPD has not been well established, and most previous reports regarding SLNB in EMPD were case reports or retrospective studies with relatively small patient numbers [94,95,96]. Fujisawa et al. conducted a multicenter retrospective study for invasive EMPD patients who underwent SLNB [7]. They found that the positive rate of SLNB in patients with invasive EMPD without lymphadenopathy was 15%, and the independent factors associated with the positive rate was invasion into the reticular dermis or deeper and presence of lymphovascular invasion [7]. From these results, they recommend SLNB with primary tumor resection for invasive EMPD patients without lymphadenopathy, although the results of SLNB did not influence survival [7]. In contrast, microinvasion may not be an indication of SLNB, as multiple studies showed that the prognosis of microinvasive EMPD was comparable with noninvasive EMPD [97]. However, it is often difficult to clinically evaluate the accurate invasion level of EMPD without histopathology obtained through primary tumor resection [95]. In addition, although rare, cases of microinvasive EMPD with lymph node metastases have been reported [98]. In some previous studies, LN metastases were also detected using SLNB in patients with microinvasive EMPD [94,95,96]. Although there has been no evidence showing that SLNB influences prognosis in EMPD patients, SLNB would provide potential prognostic information. Therefore, given that SLNB is a relatively safe procedure [7,98], simultaneous SLNB with primary tumor resection might be worth considering in EMPD cases with any clinical findings suspected of invasion, including microinvasion, such as small ulceration and slightly elevated plaques. 

### 2.7. Staging

Although EMPD usually appears as carcinoma in situ, it sometimes becomes invasive and fatal. However, a TNM staging system for invasive EMPD has yet to be established. In a multicenter retrospective study, Ohara et al. analyzed 301 patients with invasive EMPD [99]. The factors associated with survival were tumor thickness, lymphovascular invasion, number of LN metastases, and distant metastases, and from the results, they proposed the TNM and stage classification for EMPD (Table 2) [99].

In this study, patients with stage IIIa (1 LN metastasis) EMPD exhibited higher than 80% of the 5-year survival rate and showed comparable survival curve to patients with stage II, indicating that early lymphatic spread might be controlled with surgical-based treatment. On the other hand, once the tumor has spread to two or more LNs (stage IIIb), the prognosis was poor, and the 5-year survival rate dropped to 40% [99]. Therefore, establishment of effective adjuvant therapies for stage IIIb patients is needed to improve the poor survival. 

### 2.8. Evidence of Treatment

Recently, an updated version of the original Cochrane Review was published [100]. In this review, authors searched for randomised controlled trials and well-designed nonrandomised studies that compared different interventions in patients with vulvar EMPD [100]. However, none met the criteria, and most of the studies were retrospective data analyses [100]. Thus, there is a lack of a clear clinical evidence base for any EMPD treatment, and most of the treatments described below are based on previous retrospective studies and case reports. Therefore, further clinical studies of a high quality are needed to improve EMPD treatment in the future. 

### 2.9. Treatment for Primary Local Lesions

#### 2.9.1. Surgical Treatment 

Wide local excision (WLE) has long been regarded as the standard for management of EMPD. Due to the multifocal nature and discontinuous subclinical extension of Paget’s cells, most previous reports had recommended a 2 to 5 cm margin of normal skin when performing WLE [101,102,103]. However, Murata et al. analyzed 46 patients with EMPD who were surgically treated with a 1 cm margin and found that the clinically determined border of well-defined lesions of EMPD corresponded well to the histopathologic border, with the microscopic gap between the histopathological and clinical borders being 0.334 ± 1.183 mm [104]. In another study, 5 of 66 patients who underwent curative surgical excision developed local recurrence, but the surgical margin (≤2 cm or >2 cm) was not correlated with local recurrence [105]. These results suggest that a 1 to 2 cm is adequate for the surgical margin in WLE for EMPD treatment. While WLE remains the treatment of choice, the Mohs micrographic surgery (MMS) technique has gained popularity since its development in the late 1990s due to its improved recurrence rate [103]. MMS allows surgeons to microscopically examine the whole tumor margin intraoperatively [103]. However, because EMPD typically presents as a large skin lesion, MMS may be much more expensive and time consuming than WLE for such large lesions. To shorten time taken for the procedure of MMS, peripheral MMS, in which the periphery of the tumor is marked and excised until a clear margin is achieved, and its modified method have been developed [106,107].

As EMPD sometimes shows an ill-defined border or extends beyond the clinical borders, mapping biopsy has been frequently used to reduce the rate of positive surgical margin [108]. For evaluating the usefulness of the mapping biopsy in EMPD, Kaku-Ito at el performed a retrospective study of 133 patients with 150 primary EMPD lesions [109]. In this study, only 1.6% of mapping biopsy specimens from well-defined EMPD were positive. Moreover, 4.6% of mapping biopsy specimens from ill-defined EMPD were positive, whereas all specimens taken from sites of 2 cm or more from the clinical border were negative. From these results, they suggested that mapping biopsy is not required for well-defined EMPD or when 2-cm margins can be achieved. They also recommended the surgical margin to be 1 cm and 2 cm for well-defined EMPD and ill-defined EMPD, respectively. In contrast, multiple studies of patients with vulvar EMPD showed that positive margin was not associated with recurrence rate or overall survival (OS) [110,111,112,113]. This result may be explained, at least partially, by potential development of multicentric lesions in EMPD. Therefore, although negative margin would be favorable rather than positive margin, consideration for limiting the extent of resection is needed for functional or cosmetic preservation, especially in cases with resection of the urethra or anus. Irrespective of whether it is a positive or negative margin, long-term follow-up and careful examination are necessary. 

#### 2.9.2. Nonsurgical Treatment

In cases where surgery cannot be applied for reasons such as the patient’s intentions, poor general condition, or coexisting disorders, several alternative nonsurgical treatments, like radiotherapy, topical imuquimod (IMQ) cream, and photodynamic therapy (PDT), can be selected.

#### 2.9.3. Radiation Therapy

Multiple case reports demonstrated that radiation therapy was effective for primary EMPD lesions [114,115]. Hata et al. retrospectively analyzed 41 EMPD patients who underwent radiation therapy with total median dose of 60 Gy and demonstrated that the 5-year local progression-free rate was 82% [116]. In addition, no adverse events of grade 3 or more were observed, indicating that radiation therapy is safe and effective for primary EMPD lesions [116]. Given the relatively low recurrence rate, we suggest that radiotherapy should be considered as the first-choice alternative therapy to surgery. Hata et al. also analyzed 21 EMPD patients who underwent radiation therapy as postoperative adjuvant therapy and found that no patients showed local recurrence at a median follow-up period of 38 months, suggesting that radiation therapy may be effective as not only curative intent but also postoperative adjuvant therapy [117].

#### 2.9.4. Topical Imiquimod (IMQ) Cream

IMQ is a synthetic ligand of toll-like receptor 7 that has been shown to exert antitumor effects [118]. Topical 5% IMQ cream has already been approved by the US Food and Drug Administration for treatment of superficial basal cell carcinoma and actinic keratosis and has shown significant antitumor effects [118]. Previous reports also revealed antitumor response induced by topical IMQ in EMPD cases, and van et Linden et al. revealed that the numbers of CD8^+^ cells and the ratio of CD8/FoxP3 in the epidermis of vulvar EMPD were increased after IMQ treatment, suggesting that IMQ may induce a shift toward effective anti-tumor immune responses [65]. Sawada et al. prospectively evaluated topical application of IMQ 5% cream for EMPD and showed that the response rate to IMQ 5% cream for nine patients with EMPD was 100%, with 5 (56%) having achieved complete response (CR) [119]. However, three of five (60%) CR cases recurred after a long duration of CR. Therefore, although topical imiquimod is an alternative therapeutic option, long-term follow-up is needed to monitor recurrence. Machida et al. conducted a systemic literature search for vulvar EMPD treated with IMQ. In this research, 2-, 4-, and 6-month cumulative CR rates were 9.8%, 31.1%, and 71.6%, respectively, suggesting that response to IMQ treatment might be time dependent. Thus, they recommended the treatment duration to be 6 months. They also revealed that age, disease status (initial treatment or treatment for recurrence), and treatment frequency were not associated with CR rates. From these results, IMQ should be considered, especially for EMPD patients who have recurrence after multiple surgical resections [120]. Although there are some reports regarding response to IMQ in EMPD [119,120,121], it remains unclear whether response to IMQ treatment differs by gender or body region. IMQ cream might also be useful as a neo-adjuvant therapy to reduce the tumor size prior to surgery, resulting in less cosmetic and functional impairment [122]. However, caution is warranted because a retrospective study demonstrated that initial topical therapies, including IMQ cream, prior to subsequent surgery significantly increased recurrence rate [123]. Further studies are needed to better understand the features of outcomes when topical IMQ is applied in EMPD cases. 

#### 2.9.5. Photodynamic Therapy

Photodynamic therapy (PDT) is a noninvasive treatment utilizing photoreactive drugs, such as aminolevulinic acid, which are selectively taken up by tumor cells. The involved area is then exposed to the appropriate wavelength of light, creating toxic free radicals that destroy tumor cells in the process. Although multiple cases showing antitumor response with PDT have been reported, overall results suggest that PDT may not be a curative treatment but is more beneficial to be used as a palliative treatment to reduce symptoms associated with EMPD lesions [18,124,125]. The side effects of PDT include pain and photosensitivity, which could be severe in some cases [126]. 

### 2.10. Treatment for Regional LN Metastases

Although most EMPD cases display in situ lesions, dermal invasion may develop, which is frequently associated with regional LN metastases. LN dissection is a standard form of management for regional LN metastases in EMPD, although there is a lack of evidence showing that the LN dissection significantly improves overall survival in such patients. Tsutsui et al. analyzed patients with metastatic EMPD who underwent LN dissection, and their multivariate analysis revealed that the number of metastatic LNs was an independent prognostic factor for overall survival [127]. The 5-year survival rate was 100% and 19.1% in patients with two or less LN metastases and with three or more LN metastases, respectively. They also found that, in patients with three or more LN metastases, the 5-year survival rate after adjuvant radiation therapy was better than that after surgery alone, indicating that adjuvant radiation therapy for EPMD cases with three or more regional LN metastases may improve the prognosis [127].

In addition to radiation therapy for primary lesions, Hata et al. analyzed the outcomes of eight EMPD patients with pelvic and inguinal LN metastasis, representing a total of 43 metastatic LNs, who underwent radiation therapy at a total medial dose of 59.4 Gy [128]. Of the 43 metastatic LNs, only one showed progression at the median follow-up time of 22 months, and the 2-year local control rates of all metastatic LNs were 98%. From the good local control rate, radiation therapy for regional metastatic LNs might offer a curative treatment alternative to surgery and should be considered as a palliative therapy for advanced EMPD patients in whom surgical treatment is not applied. 

### 2.11. Treatment for Distant Metastases 

EMPD patients with distant metastases exhibit poor prognosis. Although conventional chemotherapies have been used for a long time in the treatment of distant metastases, there has been no prospective study showing improved overall survival with conventional chemotherapies. Hashimoto et al. conducted retrospective studies and compared the outcomes of patients with and without chemotherapy [129]. Although patients treated with conventional chemotherapies showed improved progression-free survival (PFS), OS was comparable between the two groups in their study [129]. Recent basic and clinical studies revealed multiple factors associated with EMPD as described above, and these factors could lead to new therapeutic strategies. Fukuda et al. reviewed potential treatments as well as current therapeutic approaches of advanced EMPD, including its pathogenesis [130]. 

#### 2.11.1. Chemotherapies

Although several chemotherapeutic regimens have been proposed, no standardized treatment has been established. Among the regimens, docetaxel monotherapy and low-dose 5-fluoruuracil (5-FU)/cisplatin (FP) therapy have been frequently used. Yoshino et al. conducted a multicenter, retrospective study to evaluate the efficacy of docetaxel as a first-line chemotherapy for 13 metastatic EMPD patients [131]. The disease control rate (DCR) was as high as 83%, and the median progression-free survival (mPFS), median overall survival (mOS) and 1-year OS were 7.1 months, 16.6 months, and 75.0%, respectively [131]. Tokuda et al. retrospectively examined 22 patients with advanced EMPD who received FP therapy and showed that the response rate (RR), mPFS, and mOS were 59%, 5.2 months, and 12 months, respectively [132]. Kato et al. also retrospectively analyzed eight patients with multiple metastases who were treated with FP therapy and nine patients who chose best supportive care [133]. The RR, DCR, mPFS, and mOS in patients treated with FP therapy was 50%, 75%, 6.2 months, and 19.4 months, respectively [133]. Compared with patients who received best supportive care, the mOS of the patients treated with FP therapy showed a trend towards a longer mOS, although not significant [133]. Collectively, these results suggested that the efficacy of both docetaxel monotherapy and FP therapy appeared to be comparable. Whereas myelosuppressive adverse events with grade 3 or 4 are sometimes observed in docetaxel monotherapy, FP therapy usually requires repeated hospitalization [131]. Therefore, therapy selection may be dependent on the patient’s characteristics and condition, such as age, coexisting disorders, and social circumstances. 

TS-1 is an oral chemotherapeutic drug consisting of tegaful (a prodrug of 5-FU), gimeracil, and oteracil potassium [134]. Efficacy of TS-1 monotherapy has also been reported in small number of cases [135]. Kato et al. reported two cases of advanced EMPD in which TS-1 monotherapy was effective as a second line after docetaxel treatment failure [136]. It has been reported that TS-1 plus docetaxel exhibit synergistic antitumor effects because docetaxel reduces 5-FU metabolites, resulting in increased 5-FU [137]. TS-1 has also been used in combination with docetaxel, and several case reports have demonstrated the efficacy of this combination therapy [138,139]. Recently, Matsushita et al. retrospectively analyzed 12 patients with metastatic EMPD and showed that the RR, mPFS, and mOS was 91.7%, 13.5 months, and 27.7 months, respectively [140]. This result was superior to the studies for docetaxel monotherapy and FP therapy [131,132,133]. Notably, one patient with liver metastasis who was refractory to docetaxel monotherapy responded to TS-1/docetaxel combination therapy and achieved complete response. Therefore, TS-1 plus docetaxel therapy might be more effective than docetaxel monotherapy and FP therapy, which have most commonly been used for metastatic EMPD.

As for other regimens, Oashi et al. described the efficacy of FECOM therapy, which consists of epirubicin, mitomycin C, vincristine, carboplatin, and 5-fluorouracil, in the management of seven patients with advanced EMPD and showed that the RR, mPFS, and mOS were 57%, 6.5 months, and 9.4 months, respectively [141]. Hirai et al. analyzed the outcome of PET therapy, which consists of cisplatin, epirubicin and paclitaxel, in five metastatic EMPD patients, and showed that the RR, mPFS, and mOS were 80%, 8 months, and 20 months, respectively [142]. Although these studies indicated possible efficacy of FECOM or PET therapies, both studies comprised only small number of patients; thus, further studies are required for confirmation of their findings. 

#### 2.11.2. Anti-HER2 Antibody Therapy

As described above, HER2 overexpression is frequently found in EMPD lesions, and HER2 may play crucial roles in the development and progression of EMPD in cases of HER2-positive tumors. Indeed, multiple cases of HER2-positive advanced EMPD have been reported that have shown the antitumor effects of anti-HER2 antibody, trastuzumab, monotherapy, or trastuzumab combined with other chemotherapies, such as paclitaxel or carboplatin [57,143,144,145,146,147,148]. Most of these cases showed PFS of longer than 12 months, suggesting that anti-HER2 antibody is a promising therapy for advanced EMPD. A phase II study of trastuzumab with docetaxel for HER2-positive unresectable or metastatic EMPD (UMIN00002311) has been conducted, but the results are yet to be published. 

#### 2.11.3. Hormonal Therapy

Invasive EMPD frequently shows high expression of androgen receptor (AR), indicating that AR signaling may be involved in EMPD progression, as described above. Accordingly, a case has been reported where combined androgen blockade therapy with bicalutamide and leuprolide acetate (LH-RH agonist), which were used for the treatment of prostate cancer, also improved multiple bone metastases of EMPD [149]. In a case of metastatic EMPD and metastatic prostate cancer with strong ER expression, two kinds of hormonal therapy were used: the anti-estrogen tamoxifen and the anti-androgen bicalutamide [150]. All of the metastatic lesions remained stable for 2 months after initiation of dual hormonal therapy and performance status was well maintained for 17 months, indicating that the hormonal therapies might have been beneficial for the metastases of EMPD in this case [150].

#### 2.11.4. Immune Checkpoint Therapy

Although EMPD lesions generally lack better predictors of tumor response with immune checkpoint inhibitors, such as high PD-L1/L2 expression and presence of MSI-H status, that does not mean that there would be poor anti-tumor effect from checkpoint inhibitors in all EMPD cases. Indeed, a case of metastatic EMPD with a durable response from a combination treatment of ipilimumab and nivolumab has been recently reported, although this case also showed no expression of PD-L1 or PD-L2 [151]. Further studies of advanced EMPD cases treated with checkpoint inhibitors are needed to evaluate the efficacy. Currently, a clinical trial of nivolmab and ipilimumab for treatment of rare malignancies, including EMPD (NCT02834013), is ongoing [88]. 

## 3. Conclusions

We reviewed the epidemiology, clinical presentation, classification, histopathology, pathogenesis, patient evaluation staging, and treatment of EMPD with a particular focus on recent developments. Recent basic and clinical studies provide new insights into the molecular mechanism and associated factors of EMPD development and progression. Most EMPD cases are usually diagnosed as carcinoma in situ; however, once metastases occur, EMPD frequently displays more aggressive features. Although several regimens have been proposed for the treatment of metastatic EMPD, the efficacy of conventional chemotherapies is limited. Therefore, novel therapies based on the recent basic and clinical studies that shed light on our understanding of EMPD’s pathogenesis should be developed. 

## Figures and Tables

**Table 1:** Proposed classification of vulvar Paget disease [26].

Primary Vulvar Paget Disease, a Primary Cutaneous Neoplasm	Secondary Vulvar Paget Disease
Paget disease as a primary intraepithelial neoplasm (carcinoma in situ)	Paget disease secondary to anal or rectal adenocarcinoma
Paget disease as an intraepithelial neoplasm with invasion	Paget disease secondary to urothelial neoplasm
Paget disease as a manifestation of an underlying primary adenocarcinoma of a skin appendage or a subcutaneous vulvar gland	Paget disease secondary to adenocarcinoma or related tumors of other sites

**Table 2:** Proposed TNM and stage classification system for extramammary Paget’s disease [99].

TNM			
	0	1	2
T	Tumor in situ	Tumor thickness ≤ 4 mm AND no lymphovascular invasion	Tumor thickness > 4 mm OR lymphovascular invasion
*n*	No LN metastasis	1 LN metastasis	2 or more LN metastases
M	No distant or LN metastasis beyond regional LN basin	Distant organ metastasis or LN metastasis beyond regional LN basin	(−)
**Staging**			
	T	*n*	M
I	1	0	0
II	2	0	0
IIIa	Any	1	0
IIIb	Any	2	0
IV	Any	Any	1

## Data Availability

Not applicable.
